# Risk Factors in Chronic Subdural Hematoma: Comparison of Irrigation with Artificial Cerebrospinal Fluid and Normal Saline in a Cohort Analysis

**DOI:** 10.1371/journal.pone.0103703

**Published:** 2014-08-04

**Authors:** Akihiko Adachi, Yoshinori Higuchi, Atsushi Fujikawa, Toshio Machida, Shigeo Sueyoshi, Kenichi Harigaya, Junichi Ono, Naokatsu Saeki

**Affiliations:** 1 Department of Neurosurgery, Chiba Cardiovascular Center, Tsurumai, Ichihara, Japan; 2 Department of Clinical Laboratory, Chiba Cardiovascular Center, Tsurumai, Ichihara, Japan; 3 Department of Neurological Surgery, Chiba University Graduate School of Medicine, Inohana, Chiba, Japan; 4 Department of Molecular Diagnosis, Chiba University Graduate School of Medicine, Inohana, Chiba, Japan; 5 Department of Molecular and Tumor Pathology, Chiba University Graduate School of Medicine, Inohana, Chiba, Japan; University of Michigan, United States of America

## Abstract

**Background:**

Chronic subdural hematoma (CSDH) is known to have a substantial recurrence rate. Artificial cerebrospinal fluid (ACF) is an effective irrigation solution in general open craniotomy and endoneurosurgery, but no evidence of its use in burr-hole surgery exists.

**Objective:**

To identify the potential of ACF irrigation to prevent CSDH recurrence. More specifically, to investigate the perioperative and intraoperative prognostic factors, and to identify controllable ones.

**Methods:**

To examine various prognostic factors, 120 consecutive patients with unilateral CSDH treated with burr-hole drainage between September 2007 and March 2013 were analyzed. Intraoperative irrigation was performed with one of two irrigation solutions: normal saline (NS; n = 60) or ACF (n = 60). All patients were followed-up for at least 6 months postoperatively. We also examined the morphological alternations of the hematoma outer membranes after incubation with different solutions.

**Results:**

Eleven patients (9.2%) had recurrence. Nine patients (15%) required additional surgery in the NS group, whereas only 2 patients (3.3%) in the ACF group required additional surgery. Among preoperative and intraoperative data, age (<80 years old, *P* = .044), thrombocyte (>22.0, *P* = .037), laterality (right, *P* = .03), and irrigation solution (ACF, *P* = .027) were related to smaller recurrence rates by log-rank tests. Only the type of irrigation solution used significantly correlated with recurrence in favor of ACF in both Cox proportional hazards (relative hazard: 0.20, 95% confidence interval (CI): 0.04–0.99; *P* = .049) and logistic regression models (odds ratio, 0.17, 95% CI: 0.03–0.92; *P* = .04) using these factors. Histological examinations of the hematoma membranes showed that the membranes incubated with NS were loose and infiltrated by inflammatory cells compared with those incubated with ACF.

**Conclusion:**

Irrigation with ACF decreased the rate of CSDH recurrence.

## Introduction

Chronic subdural hematoma (CSDH), typically associated with neurological deterioration after a mild head trauma in elderly patients, is one of the most common neurosurgical disorders. [1], [2] Albeit usually amenable to surgery, CSDH has been reported to have a significantly high recurrence rate, ranging from 5% to 30%. [3], [4] Although various irrigation agents such as 5% dextrose solution (D5W), normal saline (NS), lactated Ringer's solution (LR), and artificial cerebrospinal fluid (ACF) are commonly used in neurosurgery (Table 1), the role of irrigation solution has not been sufficiently assessed in the treatment of CSDH.

**Table 1:** Composition of solutions used in neurosurgery.

Component	D5W	NS	LR	ACF
Na^+^ (mEq/L)	0	154	130	145
K^+^ (mEq/L)	0	0	4	2.8
Mg^2+^ (mEq/L)	0	0	0	2.2
Ca^2+^ (mEq/L)	0	0	3	2.3
Cl^-^ (mEq/L)	0	154	109	129
HCO_3_^-^ (mEq/L)	0	0	0	23.1
P (mM/L)	0	0	0	1.1
Lactate^-^ (mEq/L)	0	0	28	0
Glucose (g/L)	50	0	0	0.61
Osmolality ratio	about 0.9	about 0.9	about 0.9	about 1
pH	about 5.0	about 6.7	about 6.7	about 7.3

Abbreviations: D5W: 5% dextrose solution, NS: Normal saline, LR: Lactated Ringer's solution, ACF: Artificial cerebrospinal fluid.

ACF is used as an irrigation fluid during neurosurgical procedures, because its composition is similar to that of human cerebrospinal fluid (CSF). [5], [6] Because of its enhanced brain-protective properties compared with conventional commercial solutions such as D5W [1], NS, [7] or LR [8], it has been used routinely, for example, in more than 1000 Japanese facilities, particularly after being marketed as a 500 mL double bag system (Artcereb, Otsuka Pharmaceutical Factory, Inc., Tokushima, Japan). [11], [12] ACF also attenuates edema around a traumatic wound, minimizes cerebrovascular permeability and cell damage, [9] and achieves faster hemostasis without interrupting normal coagulation, [10] than the other solutions.

The aim of the present study was to evaluate the relationship between the outcome of CSDH and various perioperative and intraoperative factors including the type of irrigation solution used. Here, we report on our 6-year single-center experience with CSDH, focusing on numerous factors associated with prognosis. ACF irrigation, one of the newly established prevalent factors in this study, has been known to be efficacious for intraventricular [11] or cisternal irrigation, [12] but no evidence about subdural irrigation/drainage existed. Therefore, we studied the possible beneficial effects of using ACF intraoperatively as an irrigation solution.

## Methods

### Study design

One hundred and twenty unilateral CSDH patients, who were admitted to the Chiba Cardiovascular Center for surgery (prefrontal burr-hole and overnight drainage) between September 2007 and March 2013, were analyzed in this study. All patients in this hospital cohort were symptomatic, and no patients were excluded. The first 60 patients were irrigated with NS (NS group), and the rest with ACF (ACF group).

### Ethics Statement

Before the surgery, written informed consent was obtained from all patients and/or family members. The study protocol was approved by the ethics committee of our center (Chiba Cardiovascular Center Ethical Review Board, IRB# 11000598).

### Surgical technique

Under local anesthesia and modified neuroleptanalgesia with pentazocine and diazepam, the patients underwent surgery, which included the creation of a prefrontal burr-hole, incision of the dura mater, and irrigation of the hematoma. Subdural collections were washed out with NS (n = 60) or ACF (n = 60) through a silicone catheter. Next, the subdural space was filled with either NS or ACF, and the scalp was closed in two layers. The catheter was inserted anteriorly, connected to a soft collection bag (Silascon Subdural Drainage Set, Kaneka. Medix Co., Osaka, Japan), and maintained at the head level. [8] The drainage catheter was removed the next day. Ambulation was generally allowed 3 days after the operation.

### Clinical and radiological data

Patient characteristics such as age, gender, and past medical history, including medication use, were recorded at the time of admission. Focal neurological signs including dysarthria, hemiparesis, and headache were also noted. Because a previous study identified preoperative laboratory parameters as possible predictors of CSDH recurrence, the same data sets, including preoperative platelet count, white blood cell count, and international normalized ratios of prothrombin time (PT-INR), were also collected in this study. [3] The perioperative changes in general neurological status and global disability were assessed using the Glasgow Coma Score (GCS) and modified Rankin scale (mRS), respectively. Hematoma laterality, type, [13] and volume were calculated [14] as XYZ/2 and evaluated by computed tomography (CT) at least three times in each patient: preoperatively, 1 day and 1 week (5–7 days) postoperatively, and whenever needed. All of the patients were followed-up for more than 6 months on an outpatient basis; when neurological deterioration reappeared, the patient was further evaluated using CT scans. Based on a combination of symptoms and CT findings, recurrence was defined as a state requiring reoperation for relapsed neurological symptoms with compression of the brain by an increase in hematoma volume, as relying only on symptoms or CT findings may lead to measurement and detection biases.

### Specimen Preparation

To elucidate the mechanisms underlying ACF's ability to reduce recurrence, we carried out histological exploration after the cohort analysis with 6 patients. A piece of the hematoma membrane from each patient was excised for histological examination. Each membrane (∼10 mm^2^) was cut into three pieces with a razor blade. One piece was fixed in 10% buffered formalin immediately after the surgery or after incubation in the liquid hematoma as a control. The other two pieces were fixed after different incubation times (3, 6, and 24 h) in either NS or ACF at 37°C and embedded in paraffin. These membranes were stained with hematoxylin and eosin, and then examined microscopically.

### Statistical analysis

SAS 9.4 and JMP 9 software (SAS Institute, Cary, NC) were used for statistical analyses. Continuous variables were compared between each group by using Student's *t*-tests and are reported as the mean ± the standard deviation (SD). The median values from all patients are also presented in continuous data and ordered categorical data. Fisher's exact test and χ^2^ tests were performed on proportional data. Because early readmission is associated with a poor prognosis, [15] we also used log-rank tests to evaluate the association of patient characteristics with recurrence. To perform the log-rank tests, continuous and categorical data were grouped by dichotomization with the median value.

Preoperative and intraoperative variables, which were highly associated with CSDH recurrence (*P*<.15 in the univariate analysis, *P*<.10 in the log-rank test), were subjected to further analyses to adjust for possible confounding factors. Postoperative factors were not included in the analyses to avoid multicolinearity. First, multivariate analysis was carried out using logistic regression models with these variable sets and with the propensity scores determined from these variable sets. Additionally, because of the cohort nature of this study, [16] the Cox proportional hazards model was used to assess the cumulative recurrence rate. Furthermore, we determined how sensitive the model was to the removal of the propensity regression variables. Tests for effect modifications included the addition of interaction terms for each variable to the model. For all analyses, a *P* value of <.05 was considered statistically significant. Five values of the PT-INR were missing because of incomplete routine laboratory work-ups, and these five PT-INR values were excluded from univariate analyses.

## Results

**Figure 1:**
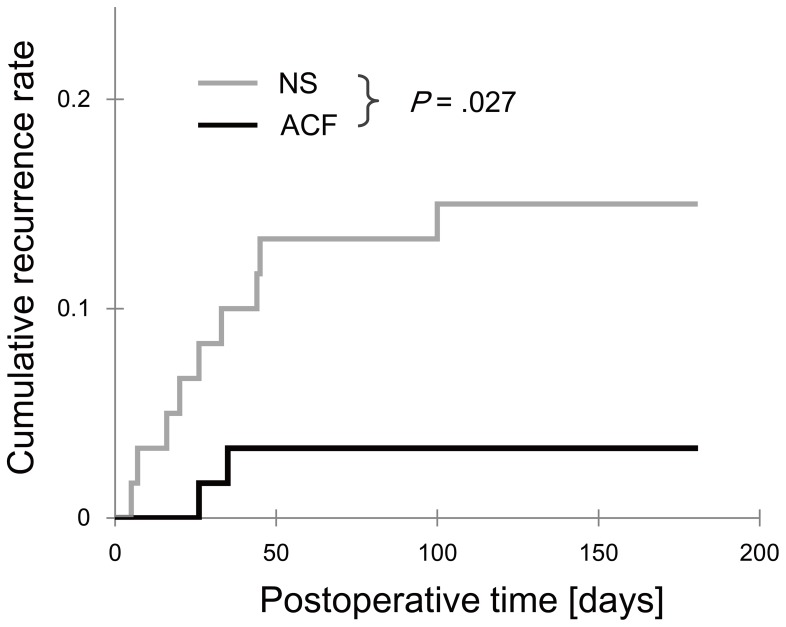
Cumulative recurrence rate of chronic subdural hematoma after surgery. The difference between the normal saline (NS) group and the artificial cerebrospinal fluid (ACF) group is statistically significant (*P* = .027, log-rank test).

Patient characteristics are listed in Table 2. The overall recurrence rate was 9.2%, with 11 patients (5 men and 6 women) out of the 120 patients experiencing recurrence during the 6 months of follow-up. Two patients (3.3%) in the ACF group and 9 patients (15%) in the NS group required additional operations (Table 3). Therefore, the use of ACF resulted in an observed crude absolute risk reduction of 11.7%. Figure 1 illustrates the postoperative cumulative recurrence rates of these patients (*P* = .027, log-rank test).

**Table 2:** Patient characteristics: median values and univariate analyses (t, U, χ^2^, and Fisher's exact tests).

		No. of patients (%)		*P* value
Factor	Median	ACF (n = 60)	NS (n = 60)	(log-rank)
Age (years)	79	77.4±10.9	76.1±12.0	.53
Age (≥80 years)		28 (47%)	25 (42%)	.092
Gender (female)		21 (35%)	23 (38%)	.34
**On admission**				
GCS 6/9/11/12/13/14/15	14	0/2/1/3/4/28/22	1/0/0/3/9/24/23	.64 (<14)
mRS 0/1/2/3/4/5	3	0/11/12/11/18/8	0/11/11/15/17/6	≥.99 (≥3)
Dysarthria		16 (27%)	12 (20%)	.51
Hemiparesis		57 (95%)	53 (88%)	.32
Headache		17 (28%)	23 (38%)	.33
**Past history**				
Cerebrovascular accident		14 (23%)	9 (15%)	.35
Diabetes mellitus		9 (15%)	14 (23%)	.35
Hypertension		31 (52%)	32 (53%)	≥.99
Heavy drinking		14 (23%)	9 (15%)	.35
Ischemic heart disease		7 (12%)	5 (8%)	.76
Tumor		7 (12%)	6 (10%)	≥.99
Drug history (anticoagulant)		7 (12%)	6 (10%)	≥.99
Drug history (antiplatelet)		21 (35%)	13 (22%)	.16
**CT findings**				
Laterality (left)		29 (48%)	32 (53%)	.72
Hyper/Iso/Hypo/Layer/Grad/Sep/Trab		12/9/5/8/5/7/14	12/5/7/8/6/8/14	.79–≥.99
Hematoma cavity volume (mL)	137	147±49	149±49	.76
Preoperative laboratory data				
Leukocytes, ×10^9^/L	6.60	7.21±2.77	7.01±2.68	.69
Thrombocytes, ×10^9^/L	220	250±85	209±103	**.02**
PT-INR	1.00	1.02±0.096	1.00±0.095	.11
**Complications**				
Transient postoperative delirium		12 (20%)	11 (18%)	≥.99
Urinary tract infection		3 (5%)	1 (2%)	.62
**Postoperative data**				
GCS (10/12/13/14/15)	15	0/1/1/16/42	1/1/0/18/40	.84 (<14)
mRS 0/1/2/3/4/5	1	30/10/4/7/9	23/15/5/8/8/1	≥.99 (≥3)
Cavity volume (mL) on POD 1	63.9	71±32	69±40	.83
Cavity volume (mL) on POD 7	62.8	59±37	74±40	**.028**
Hospital stay (days)	8	9.5±4.0	10.5±7.2	.37

Values are the mean ± SD when appropriate. Significant values are featured with bold font. Three PT-INR values from the NS group (1 recurrent patient) and 2 PT-INR values from the ACF group were missing and were excluded from analysis. Abbreviations: ACF: Artificial cerebrospinal fluid, NS: Normal saline, GCS: Glasgow Coma Score, mRS: Modified Rankin scale, CT: Computed tomography, Hyper: Hyperdense, Iso: Isodense, Hypo: Hypodense, Layer: Layered, Grad: Gradation, Sep: Separated, Trab: Trabecular, PT-INR: International normalized ratio of prothrombin time, POD: Postoperative day.

**Table 3:** Factors related to recurrence: univariate analyses (t, U, χ^2^, and Fisher's exact tests) and log-rank tests.

	No. of patients (%)		*P* value	*P* value
Factor	No rec (n = 109)	Rec (n = 11)	(univariate)	(log-rank)
Age (years)	76.3±11.7	75.1±6.1	**.025**	**.044** (≥80 y)
Gender (female)	38 (35%)	6 (55%)	.34	.20
**On admission**				
GCS 6/9/11/12/13/14/15	1/2/1/6/9/45/45	0/0/0/0/4/7/0	.26 (<14)	.13 (<14)
mRS 0/1/2/3/4/5	0/21/21/25/29/13	0/1/2/1/6/1	.20 (≥3)	.48 (≥3)
Dysarthria	23 (21%)	5 (46%)	.13	.067
Hemiparesis	99 (91%)	11 (100%)	.63	.31
Headache	38 (35%)	2 (18%)	.33	.26
**Past history**				
Cerebrovascular accident	19 (17%)	4 (36%)	.22	.12
Diabetes mellitus	21 (19%)	2 (18%)	≥.99	.93
Hypertension	56 (51%)	7 (64%)	.65	.44
Heavy drinking	21 (19%)	2 (18%)	≥.99	.91
Ischemic heart disease	12 (11%)	0 (0%)	.60	.26
Tumor	12 (11%)	1 (9.1%)	≥.99	.84
Drug history (anticoagulant)	12 (11%)	1 (9.1%)	≥.99	.84
Drug history (antiplatelet)	31 (28%)	3 (27%)	≥.99	.93
**CT findings**				
Laterality (left)	52 (48%)	9 (82%)	.067	**.03**
Hyper/Iso/Hypo/Lay/Grad/Sep/Trab	23/15/9/15/10/13/24	1/1/1/1/1/2/4	.28–≥.99	.26–.96
Hematoma cavity volume (mL)	147±48	158±53	.45	.36
Preoperative laboratory data				
Leukocytes (×10^9^/L)	7.2±2.8	6.4±2.2	.39	.33 (>6.6)
Thrombocytes (×10^9^/L)	234±97	186±80	.12	**.037** (≤22.0)
PT-INR	1.01±0.09	1.03±0.12	.53	.80 (≥1.00)
**Intraoperative irrigation solution**				
NS versus ACF	58 (53%)	2 (18%)	.053	**.027**
**Complications**				
Transient postoperative delirium	19 (17%)	4 (36%)	.22	.10
Urinary tract infection	4 (3.7%)	0 (0.0%)	≥.99	.53
**Postoperative data**				
GCS on POD 5, 10/12/13/14/15	1/1/1/18/78	0/1/0/6/4	≥.99 (<14)	.19 (<14)
mRS on POD 5, 0/1/2/3/4/5	51/21/7/14/15/1	2/4/2/1/2/0	≥.99 (≥3)	.94 (≥3)
Cavity volume (mL) on POD 1	69±36	79±32	.41	.74 (≥64)
Cavity volume (mL) on POD 7	63±38	97±31	**.005**	**.044** (≥63)
Hospital stay (days)	9.8±5.3	11.6±10.2	.57	.81

Values are the mean ± SD when appropriate. Significant values are featured with bold font. Four PT-INR values from the non-recurrent group and 1 PT-INR value from the recurrent group were missing and were excluded from analysis. Abbreviations: Rec: Recurrence, GCS: Glasgow Coma Score, mRS: Modified Rankin scale, CT: Computed tomography, Hyper: Hyperdense, Iso: Isodense, Hypo: Hypodense, Lay: Layered, Grad: Gradation, Sep: Separated, Trab: Trabecular, PT-INR: International normalized ratio of prothrombin time, NS: Normal saline, ACF: Artificial cerebrospinal fluid, POD: Postoperative day.

Five preoperative PT-INR values did not exist (missing completely at random because of simple measuring loss by the primary doctor). Therefore, we restricted the univariate analysis of PT-INR to the 115 patients that had PT-INR values. The results generated using imputation methods did not differ substantively from the results generated by this case-wise deletion. All other variables were complete.

Among the preoperative and intraoperative factors, age (<80 years old, *P* = .044), irrigation solution (ACF, *P* = .027), preoperative platelet count (>22.0, *P* = .037), and laterality (right, *P* = .030) were related to a low recurrence rate with the log-rank test (Table 3). None of the symptoms, such as dysarthria (*P* = .067), showed a statistically significant correlation with recurrence. The only significant factor was the type of irrigation solution used, indicating the effectiveness of ACF (Table 4) in the Cox proportional hazards models. Additionally, logistic regression models using these 5 factors (Table 4) or the propensity score (odds ratio of ACF, 0.18, 95% CI: 0.036–0.94; *P* = .041) showed similar results. The ACF odds ratio changed only .03 when any single variable was removed. No effect modification was detected in these variables (*P* for interaction >.53).

**Table 4:** Multivariate analyses of chronic subdural hematoma recurrence^a^.

	Logistic regression analysis			Cox proportional hazards model		
Factor	OR	95% CI	*P*	Hazard ratio	95% CI	*P*
ACF	0.17	0.03–0.92	**.04**	0.20	0.04–0.99	**.049**
Age	1.08	0.98–1.18	.12	1.06	0.98–1.15	.06
Dysarthria	4.06	0.91–18.1	.068	3.10	0.90–10.7	.074
Laterality (left)	3.39	0.62–18.5	.16	3.15	0.63–15.8	.16
Thrombocyte	0.98	0.90–1.07	.71	0.98	0.92–1.06	.75

Significant values are featured with bold font. Abbreviations: OR: Odds ratio, CI: Confidence interval, ACF: Artificial cerebrospinal fluid.

Amidst the postoperative data, a sharp decrease in the residual subdural cavity volume was observed in CT images on postoperative day (POD) 1 in both groups; however, on POD 5–7, the cavity volume in the ACF group was less than that in the NS group (*P* = .02; Table 2).

### Histological alterations in the hematoma membrane

**Figure 2:**
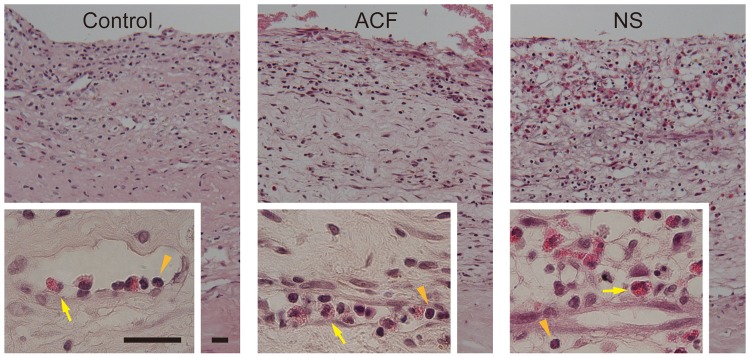
Cross sections of the hematoma outer membranes fixed immediately after surgery (Control, left panels) or after 24 h of incubation with artificial cerebrospinal fluid (ACF, center panels) or normal saline (NS, right panels). The insets are higher magnifications of the perivascular area. Inflammatory cells, including lymphocytes (orange arrowheads) and eosinophils (yellow arrows), are mostly located in the loose stroma of the hematoma membrane in the NS group, whereas they are confined in the capillary lumen, in the control and ACF groups. Hematoxylin–eosin stain, Burr = 25 µm.

**Figure 3:**
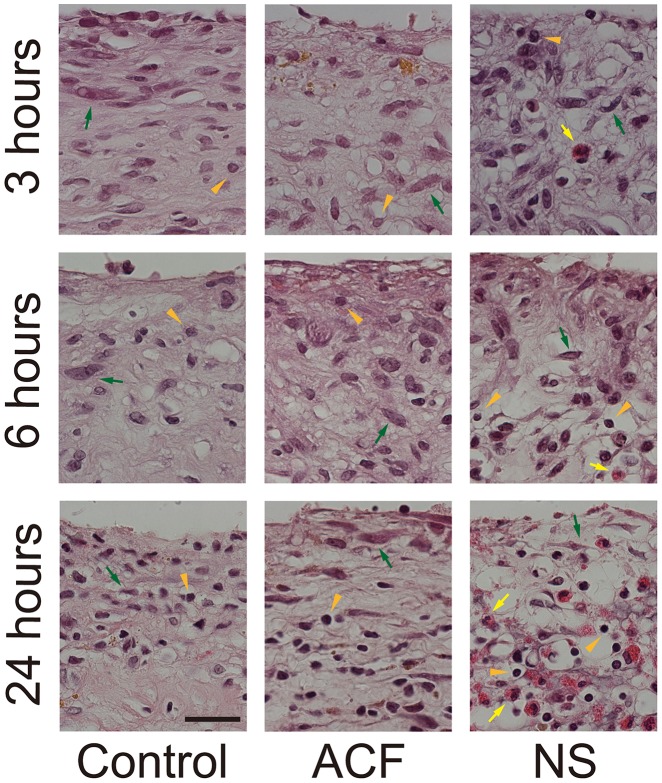
The membranes were fixed after 3, 6, or 24 h of incubation. Fewer morphological changes are seen in the control (left panels) or ACF groups (center panels), whereas the cytoplasm of constituent cells shrank and the nuclei of most infiltrated cells are pyknotic and deformed (round) in the NS group (right panels). The former alteration is the most noticeable in fibroblasts (green arrows) and the latter in lymphocytes (arrowheads).

We observed a striking disorganization with increased luminosity in the hematoma membranes incubated in NS compared to the control or to the membranes incubated in ACF (Figures 2 and 3). Microscopic analysis revealed the loss of extracellular matrix, shrinkage of the cytoplasm, and pyknotic change of nuclei in the outer membranes treated with NS. The chromatin structure was maintained both in the control and in ACF-incubated groups (Figure 3). Eosinophils infiltrated the loose membrane interstitium in the NS group, whereas they were mostly within the microvascular lumens in the control and ACF groups (Figure 2).

## Discussion

Several factors are known to increase the risk of CSDH recurrence following burr-hole drainage surgery. However, the involvement of the intraoperative irrigation solution in the recurrence of CSDH has not been examined sufficiently. We identified one prospective randomized study on the effect of ACF on cerebral blood flow and favorable postoperative status after clipping of unruptured cerebral aneurysms, [12] and one retrospective case-control study on the effect of minimizing inflammation after an endoscopic procedure for aqueduct stenosis. [11] Here, we showed that patients with ACF irrigation had a lower recurrence rate than those receiving NS irrigation. No previous studies have explored the use of ACF in burr-hole drainage surgery in CSDH patients to decrease the residual hematoma volume and CSDH recurrence rate.

The space irrigated in this study was subdural rather than subarachnoidal or intraventricular, where CSF is usually located. However, a tear in the subarachnoid membrane has been verified as a cause of CSDH. [17] Furthermore, even without the tear, irrigation fluid is known to influence the arachnoid mater and the underlying brain parenchyma. [18] Thus, we postulated that ACF, as compared with NS, would minimize irritation, improve the stability and permeability of the hematoma membrane, and promote another healing process, i.e., the physiological re-expansion of the brain, allowing cavity volume reduction and preventing recurrence.

Since Virchow initially described CSDH as “pachymeningitis haemorrhagica interna,” the etiology of CSDH has long been considered to include inflammation. [19], [20] In our study, ACF (buffer solution with pH 7.3), which can be less irritating than NS (simple salt solution with pH 6.3), was shown to decrease the damage to the hematoma membranes and the hematoma volume, thus decreasing the recurrence (Table 2, Figure 1). Based on these theoretical rationales and our clinical data, the use of ACF as an irrigation solution could be beneficial, not only for subarachnoid or intraventricular lesions, but also for subdural lesions.

The primary limitation of this study was that it involved a single advanced medical center. Nevertheless, our hospital cohort included a broad range of patients, from adolescents to elderly individuals and both mild and severe cases, which ensures the generalizability of the results. Further, as our public center is located in a rural area, it also functions as the sole community hospital. In the future, a multicenter parallel group comparison study is necessary for evaluating the specific effects and power of the recurrence reduction in a more general population. This preliminary study suggests that additional rigorous investigations are warranted to determine whether ACF is beneficial for reducing the risk of recurrent CSDH. Currently, two prospective studies are ongoing. [21], [22]

## Conclusions

In the present study, which consisted of 120 consecutive patients, we investigated risk factors related to the recurrence of CSDH and verified the independent role of irrigation solution in CSDH recurrence. Based on our findings, we conclude that irrigation with ACF is associated with a decreased incidence of recurrence.
